# Awareness of Stroke and Health-seeking Practices among Hypertensive Patients in a Tertiary Care Hospital: A Cross-sectional Survey

**DOI:** 10.7759/cureus.4774

**Published:** 2019-05-28

**Authors:** Nayab Z Dar, Shahzad A Khan, Arsalan Ahmad, Shereen Maqsood

**Affiliations:** 1 Miscellaneous, Shifa International Hospital, Islamabad, PAK; 2 Miscellaneous, Health Services Academy, Islamabad, PAK; 3 Neurology, Shifa International Hospital, Islamabad, PAK

**Keywords:** stroke, awareness, practices, hypertension, pakistan

## Abstract

Introduction: Stroke is a major cause of death with hypertension being identified as an important modifiable risk factor. Prompt identification of stroke symptoms and timely management is noted to be significant in lowering both morbidity and mortality. Baseline stroke knowledge in hypertensive patients is crucial to develop effectively targeted, and appropriate health promotion campaigns; thus, the main objectives of this study are to assess the awareness of stroke and to determine health-seeking practices among hypertensive patients.

Materials and methods: A standardized questionnaire survey regarding awareness and practices about stroke among hypertensive patients was conducted in a tertiary care hospital of Islamabad. The sample size was calculated as 384.

Results: Out of 384 patients evaluated, 80.5% had heard about stroke, 71.6% knew someone with stroke, and 76% identified the brain as the organ affected. Sudden onset numbness of limb (66.9%) and hypertension (93.5%) were common warning symptom and risk factor identified. 87.5% would take stroke patients to a hospital. Only 45.1% of the patients took their medications regularly, and 38% checked their blood pressure.

Conclusion: Majority of hypertensive patients were aware of stroke but the awareness of risk factors and warning signs was poor. Stroke prevention practices were also sub-optimal. There is a need to increase knowledge regarding risk factors, which will benefit the community at large.

## Introduction

Stroke is a major cause of death worldwide and the most common cause of death in low- and middle-income countries [1]. Early identification of stroke symptoms and appropriate, timely management can significantly reduce both morbidity and mortality [2-3]. Stroke patients in developing countries such as Pakistan are almost a decade younger than in western countries, leading to greater disability and economic losses [4]. More than 90% of the burden of stroke is linked to modifiable risk factors including behavioral, metabolic, and environmental factors [5]. Risk factors for stroke range from diabetes mellitus, hypertension, hyperlipidemia, atrial fibrillation, aneurysms, arterio-venous malformation, and smoking [6-7]. Hypertension has consistently been recognized as a crucial modifiable risk factor for stroke [8].

The prevention of stroke has not only proven to be practicable, but it has also been shown to be effective. Approximately 80% of strokes may be prevented when necessary precautions and actions are taken that are derived from knowledge of the disease’s risk factors [8]. Prevention of stroke is highly influenced by the level of awareness of individuals and the community regarding the identification of stroke risk factors and subsequent management of stroke symptoms. If done timely, the primary preventive measures and medical attention could be lifesaving.

Baseline stroke knowledge in hypertensive patients is crucial to develop effectively targeted, and appropriate health promotion campaigns to prevent stroke. The main objectives of this study are to assess the awareness of stroke in hypertensive patients, to assess health-seeking practices among hypertensive patients, and to determine the association of socio-demographic variables, warning signs, and risk factors identified with practices of hypertensive patients.

## Materials and methods

This study was approved as part of the thesis for Master of Science in Public Health of Health Services Academy, Islamabad and was approved by the Internal Review Board committees of Health Services Academy and Shifa International Hospital. This study was a cross-sectional survey and was conducted in three months from May 2018 to July 2018. Participants were hypertensive patients visiting outpatient department clinics. It was conducted in Shifa International Hospital, a tertiary care hospital of Islamabad. This hospital offers outpatient facilities in different specializations. It also has a welfare clinic adjacent to the hospital, and it caters to deserving patients daily. Hypertensive patients from both private and welfare clinics were enrolled to get a broader variety of patients with different socio-economic backgrounds.

The data collection tool was a questionnaire from a knowledge, attitude, and practice (KAP) study in Nepal of stroke [9]. The structured questionnaire consisted of 15 questions and was developed by a group of medical professionals (nurses and doctors trained by a neurologist) and was pretested before the data collection process. 

The questions were designed to cover KAPs with respect to stroke and had “yes” or “no” responses. After permission had been granted from the corresponding author, the questionnaire was adapted for our purposes. Questions related to the self-care practices of the hypertensive patients were added to the questionnaire. Questions related to practices upon witnessing a person with stroke symptoms were also included. Questions related to attitude towards stroke were excluded.

A consultant neurologist was consulted about the face validity of the questionnaire for this study as well. Forward and back translation of the questionnaire was done in Urdu to validate the questionnaire after it was adapted. The questionnaire was pre-tested in a group of 30 hypertensive patients before the start of the survey. The questionnaire has been presented in the Appendix.

All patients diagnosed with hypertension were included, and any patients with prior history of any stroke and transient ischemic attacks were excluded. Individuals unable to physically undergo an interview or with intellectual disabilities were also excluded. Patients were approached by the researcher during the initial assessment in outpatient clinics. After informed consent was provided, patients were debriefed and given a simple, clear, and informative explanation of the rationale of the study and the questionnaire. They were interviewed using a structured, standardized questionnaire. No attempt was made to prompt the participants by suggesting answers.

Non-probability consecutive sampling was done in Falahi, nephrology, medicine, and cardiology outpatient departments until the adequate sample size was achieved. The sample size calculation for stroke awareness was based on the prevalence of hypertension which is a major risk factor for stroke. A previous study showed hypertension prevalence as 35.1% [10]. The sample size was calculated by the following formula: n = z2*p(1-p)/e2. At a prevalence rate of .35, an error rate of .05 and a z value of 1.96, the required sample size was 349. 10% was added for refusals. The total sample size calculated was 384 participants.

Data were analyzed using IBM SPSS Statistics for Windows, Version 22.0. (IBM Corp., Armonk, NY). Basic descriptive statistical analysis was done including counts and means. Chi-square test with p-values (traditional cut off for significance: p <0.05) was applied to determine the association of awareness, risk, warning signs identified, and health-seeking practices.

## Results

A total of 384 hypertensive patients were interviewed using the standardized questionnaire. We interviewed (52.9%) male patients and 181 (47.1%) female patients with a mean age of 51.4 years. Most patients were Muslim (92.4%), followed by Christians (6%) and Hindus (1.6%) (Table 1).

**Table 1:** Sociodemographic characteristics

	Characteristic	N (%)
Age (years)	30-39	53 (13.8%)
	40-49	120 (31.3%)
	50-59	125 (32.6%)
	60-69	62 (16.1%)
	>70	24 (6.2%)
Gender	Male	203 (52.9%)
	Female	181 (47.1%)
Religion	Muslim	355 (92.4%)
	Christian	23 (6%)
	Hindu	6 (1.6%)
Income	Lower	61 (15.9%)
	Middle	207 (53.9%)
	Higher	116 (30.2%)
Education	Illiterate	34 (8.9%)
	Primary	51 (13.3%)
	Secondary	58 (15.1%)
	Higher	241 (62.8%)
Marital Status	Single	52 (13.5%)
	Married	332 (86.5%)

Two hundred seven (53.9%) patients belonged to middle-income status while 61 (15.9%) were from lower income status; 116 (30.2%) were from higher income status. More than half of the patients had higher education (62.8%) while 34 (8.9%) patients were illiterate; 51 (13.3%) had primary education, and 58 (15.1%) had secondary education (Table 1). Two hundred seventy-five (71.6%) patients personally knew someone with stroke while 194 (50.5%) patients reported having a family history of stroke.

Most patients (80.5%) had heard or read about stroke. Two hundred ninety-two (76%) patients correctly identified stroke as a disease of the brain. One hundred twenty-six (32.6%) patients believed that stroke is a disease limited to the elderly population while 168 (43.8%) believed stroke was a hereditary disease. We noted several misconceptions of the disease state, such as patients reported a belief that stroke is the result of an ancestor’s sin (27.3%) or that stroke is a contagious disease (22.9%). Most of the patients (85.4%) thought that the stroke could be treated and prevented. However, 167 (43.5%) patients felt that homeopathic treatment is effective (Table 2).

**Table 2:** Positive responses of hypertensive patients' awareness and practices

	Questions	N (%) (Positive Responses)
Awareness	Have you ever read/heard about a disease called stroke?	309 (80.5%)
	Is stroke a disease of the brain?	292 (76%)
	Is stroke contagious?	88 (22.9%)
	Is stroke an old person disease?	126 (32.6%)
	Is stroke a hereditary disease?	168 (43.8%)
	Do you think stroke is caused by ancestor’s sin?	105 (27.3%)
	Do you think stroke can be prevented?	328 (85.4%)
	Do you think homeopathic treatment is beneficial for stroke?	167 (43.5%)
Practices	Do you:	
	Exercise:	188 (49%)
	Consume fruits and vegetables	292 (76%)
	Avoid fatty foods	155 (40.4%)
	Take medicines regularly	173 (45.1%)
	Check your blood pressure regularly	146 (38%)
	Avoid smoking	327 (85.2%)
	What will you do if you happen to witness a person with stroke?	
	Take him/her to the hospital	326 (87.5%)
	Sprinkle water on face	28 (7.3%)
	Wait for spontaneous recovery	17 (4.4%)

Most patients (76%) consumed fruits and vegetables while around half of the patients (49%) exercised. However, 40.4% of the patients avoided fatty foods; 45.1% of the patients regularly took their medications while the majority of the patients (85.2%) avoided smoking. Three hundred thirty-six (87.5%) patients, upon witnessing a person exhibit stroke symptoms, stated they would take that person to a hospital in an emergency, while 28 (7.3%) reported they would sprinkle water over the face of a person having a stroke, and 17 (4.4%) said they would wait for spontaneous recovery (Table 2).

The mean number of risk factors identified were 2.6. Most of the patients identified one or more risk factors (97.7%) while only 19.5% identified all the provided risk factors; 2.3% of the patients did not identify any of the provided risk factors. The mean number of warning signs identified was 1.5. Most (73.7%) patients identified at least one symptom while 26.3% identified none of the warning signs. None of the respondents could identify all the provided warning symptoms (Table 3).

**Table 3:** Number of risk factors and warning signs identified by hypertensive patients

	Categories	N (%)
Risk Factors	Zero	9 (2.3%)
	One	92 (24%)
	Two	102 (26.8%)
	Three	73 (19%)
	Four	32 (8.3%)
	Five	75 (19.6%)
Warning Signs	Zero	101 (26.2%)
	One	99 (25.7%)
	Two	99 (25.7%)
	Three	51 (13.7%)
	Four	18 (4.6%)
	Five	16 (4.1%)
	Six	0
	Seven	0

**Figure 1:**
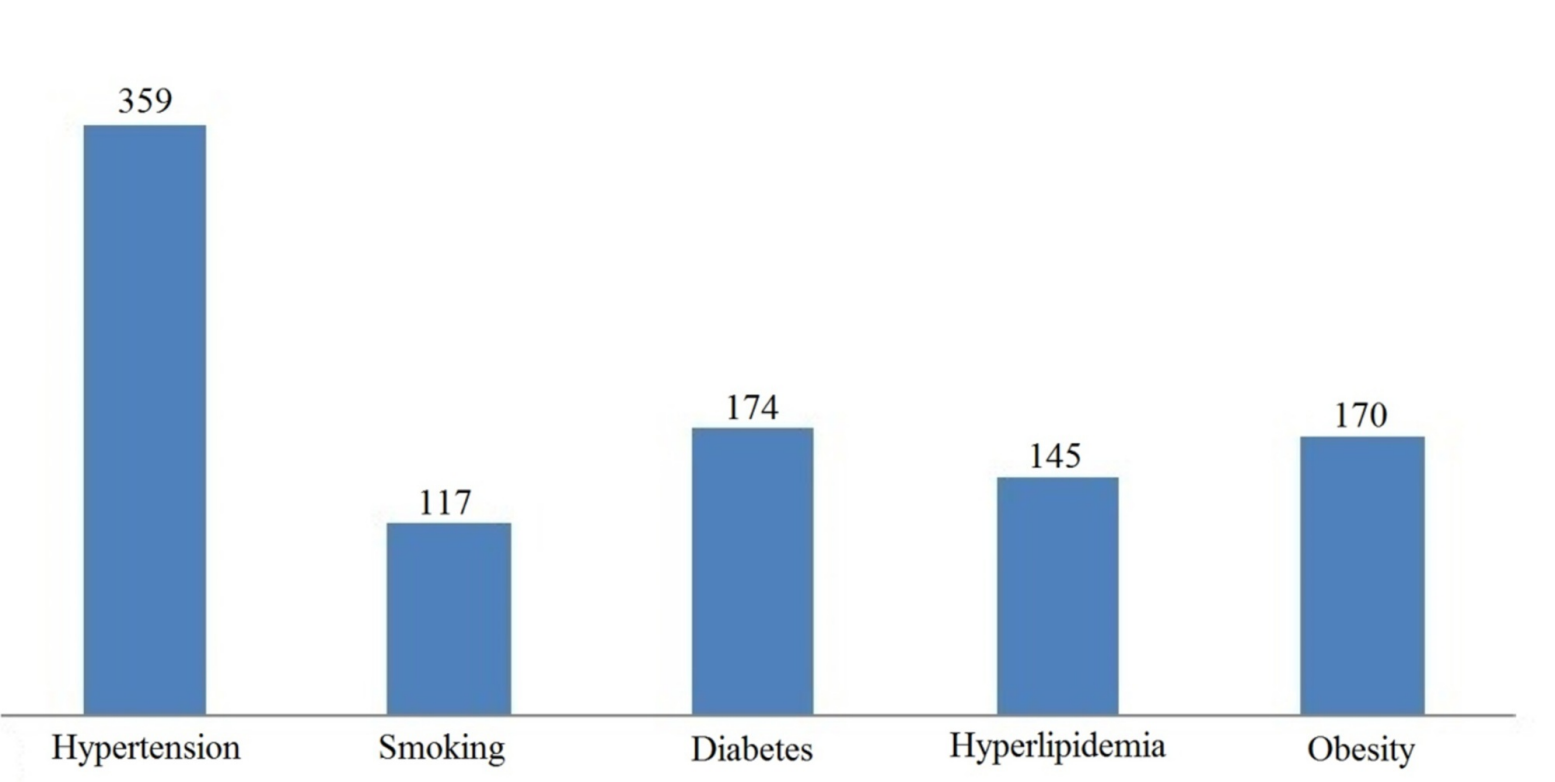
Risk factors of stroke identified by hypertensive patients

A majority (93.5%) of patients identified hypertension as a risk factor for stroke followed by diabetes (45.3%), smoking (44.8%), obesity (44.3%) and hyperlipidemia (37.8%) (Figure 1).

**Figure 2:**
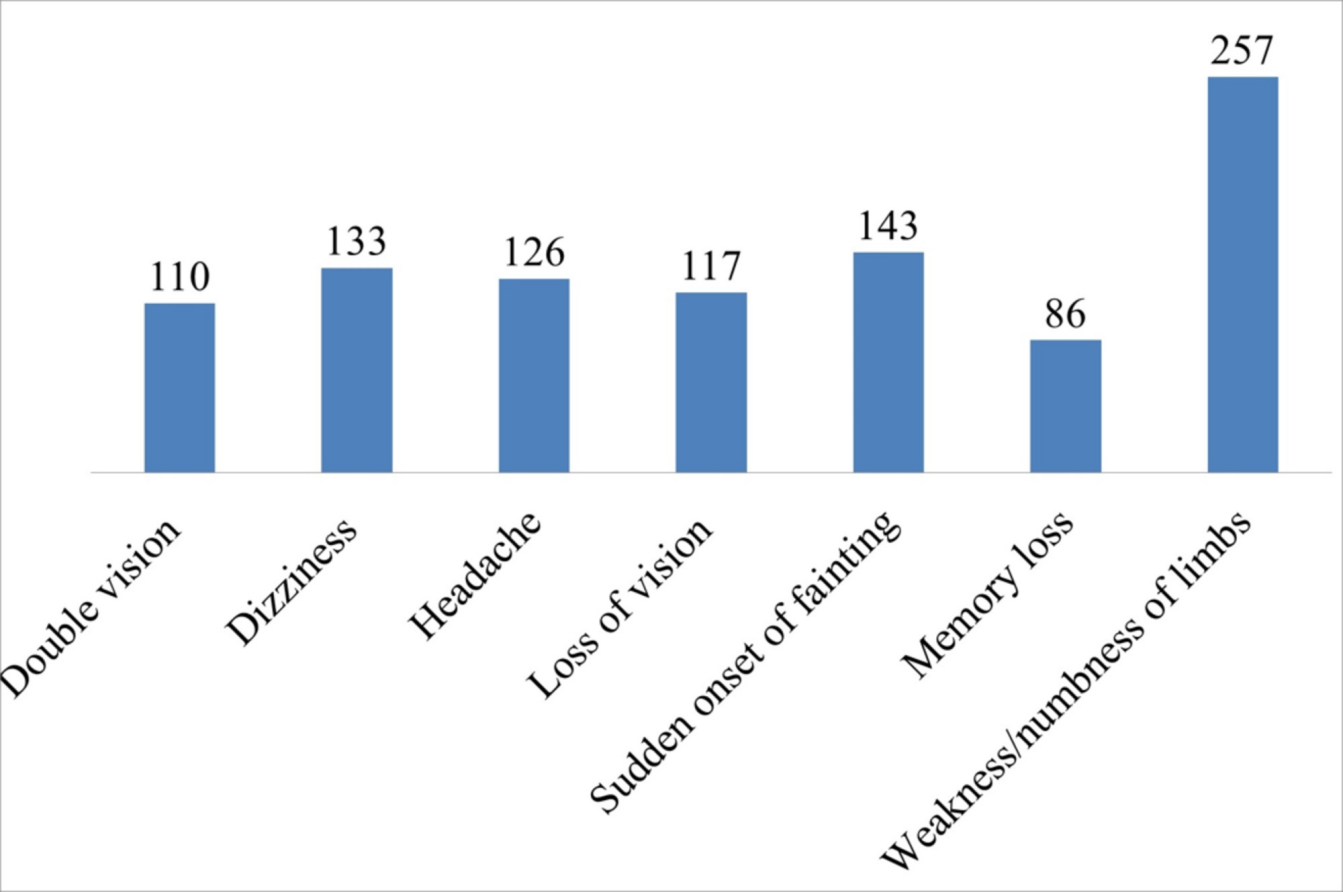
Warning signs of stroke identified by hypertensive patients

Sudden onset of weakness/numbness of limbs (66.9%) and fainting (37.2%) were the most commonly identified warning sign/symptom followed by sudden onset of dizziness (34.6%), sudden onset of headache (32.8%), sudden onset of loss of vision (30.5%), sudden onset of double vision (28.6%) and sudden onset of memory loss (22.4%) (Figure 2).

The Chi-square test showed no significant association of age, gender, religion or family history of stroke, personally knowing someone with stroke with at least identifying one risk factor or warning symptom (Table 4).

**Table 4:** Risk factors and warning signs identified according to demographic characteristics

		≥1 Risk factors Identified			≥1 Warning signs Identified		
		Yes	No	p	Yes	No	P
Age (years)	30-39	51	2	0.877	40	13	0.452
	40-49	118	2		95	25	
	50-59	112	3		87	38	
	60-69	61	1		45	17	
	>70	23	1		16	8	
Gender	Male	197	6	0.311	152	152	0.333
	Female	176	3		131	131	
Religion	Muslim	347	8	0.756	257	98	0.103
	Christian	22	1		20	3	
	Hindu	6	0		6	0	
Income	Lower	58	3	0.284	45	16	0.295
	Middle	204	3		153	54	
	Higher	113	3		85	31	
Education	Illiterate	32	2	0.391	25	9	0.992
	Primary	49	2		32	19	
	Secondary	57	1		44	14	
	Higher	237	4		182	59	
Marital Status	Single	40	3	0.109	20	12	0.351
	Married	326	6		243	89	
Family History of Stroke	Yes	191	3	0.241	148	46	0.249
	No	184	6		135	55	
Personally Know Someone with Stroke	Yes	270	5	0.232	198	77	0.249
	No	105	4		85	24	​​​​​​​

However, there was significant association of choosing to take a patient to a hospital (if they witness him/her with stroke) with higher income (P <0.001), higher education (P<0.001), at least identifying one risk factor (P=0.002), and personally knowing someone with stroke (P<0.001) (Table 5).

**Table 5:** Willingness to take a stroke patient to the hospital by demographics, knowledge, and practice

		Taking a person to the Hospital		
		Yes	No	P
Age (years)	30-39	50	3	0.128
	40-49	107	13	
	50-59	108	17	
	60-69	49	13	
	>70	22	2	
Gender	Male	180	23	0.281
	Female	156	25	
Religion	Muslim	312	43	0.259
	Christian	18	5	
	Hindu	6	0	
Income	Lower	45	16	<0.001
	Middle	195	12	
	Higher	96	20	
Education	Illiterate	18	16	<0.001
	Primary	42	9	
	Secondary	45	13	
	Higher	231	10	
Marital Status	Single	44	8	0.314
	Married	292	40	
Risk factors identified ≥1	Yes	332	43	0.002
	No	4	5	
Warning Signs identified ≥1	Yes	248	35	0.509
	No	88	13	
Family History of Stroke	Yes	172	22	0.295
	No	164	26	
Personally Know someone with Stroke	Yes	252	23	<0.001
	No	84	25	​​​​​​​

## Discussion

Pakistan is a developing country and is doing its best to improve the state of its health sector with the help of public and private hospitals. We are in the era of interventional treatment of stroke with the practice of thrombolysis and mechanical thrombectomy now being steadily introduced. In Pakistan, these interventions require state of the art facilities that carry a high expense and are only available in a few private hospitals. Tissue plasminogen activator, as a registered medical product by the Ministry of Health Pakistan, has extremely limited availability. Efforts are being made by the neurologist community in Pakistan to address this lack of availability [11]. However, before thrombolysis can be the norm for treatment for acute stroke, public understanding of stroke and its risk factors and warning signs has to improve.

In this hospital-based survey, more than two-thirds of patients had heard or read about stroke. We found poor knowledge of risk factors and warning signs, and inadequate practice with respect to stroke. Similarly, another study in Kottayam reported that of 60 selected patients, 47 patients had inadequate knowledge, 13 had moderate knowledge, and none had sufficient knowledge regarding stroke prevention [12].

While we found no significant association between gender and knowledge of risk factors or warning signs, male respondents identified risk factors and warning signs more than female respondents. This is similar to the results in a study on high school students in Nepal. However, no consensus exists on a significant difference in knowledge by gender [9].

This study was done in patients with different education levels, yet we found no significant association between education level, risk, and warning signs identified. This indicates that patients do not receive any extra knowledge regarding stroke, and educational campaigns about stroke might improve understanding and behavior regarding stroke.

Of all respondents, 71.6% of patients reported personally knowing someone with stroke, and half of the patients reported a family history of stroke. Closer interpersonal and interfamily relationships in developing countries like Pakistan can be related to the high degree of familiarity with stroke. Having a family history of stroke or personally knowing someone with stroke was not associated with having knowledge about stroke risk factors or warning signs. This signifies a need to educate the population about the family history as an important risk factor for stroke.

About one-fourth of the patients did not recognize stroke to be a disease of the brain. This is similar to results obtained in a study done in Nigeria [8]. Several misconceptions about the cause of stroke were identified in our study. Of those surveyed, 43.8% of patients believed stroke to be a hereditary disease, and a few respondents believed stroke to be a contagious disease or associated with an ancestor’s sin. These results suggest that Pakistan needs to encourage educational programs regarding stroke to increase the understanding of the general population and reduce the misconceptions.

Nearly all respondents (93.5%) identified hypertension to be a risk factor for stroke. Diabetes and obesity were the subsequent risk factors identified. However, a systematic review concluded Indian stroke survivors, as well as the general public, identify diabetes as a significant factor [13]. An even higher proportion of patients (97.1%) identified one or more risk factors while only 19.5% identified all the provided risk factors. This is in contrast with a study done in India, where it was observed that the general population, 32% of men, and 28% of women recognized more than one stroke risk factor [14].

Interestingly, about 72% of the patients believed that stroke can present with sudden weakness or numbness of limbs. Similarly, a study reported the highest symptom which was accurately recognized was hemiplegia/hemianesthesia (14.8%) followed by speech deficit (10.3%) in a study done among stroke caregivers [4].

Risk factor and warning sign awareness vary, but it indicates the necessity for the overall improvement of the existing awareness not limited to a single particular risk factor or warning sign. Most patients (85.4%) believed stroke could be prevented. However, 43.5% of the patients believed homeopathic medication could treat stroke. This can be due to the strong influence of homeopathic medication in developing countries.

Patients with good knowledge about risk factors tended to choose to take a stroke patient to a hospital and were more likely to have better income, higher education than other respondents and could identify one or more risk factors as well as know someone with stroke. This is similar to a study done in Argentina, in which, compared to lower levels of education, those with higher levels of education were more likely to seek emergency services as appropriate [15]. This indicates that increasing the awareness of risk factors and warning signs of stroke increase the awareness of patients to seek emergency medical services.

Most patients in this study stated that they consumed fruits and vegetables while only 40.4% of the patients avoided fatty foods. In contrast, a study done in India stated that only 37.1% of the hypertensive patients had a regular intake of fruits and vegetables [16].

This study did have a few limitations. This was a single-center study conducted in an urban setting and therefore is not representative of a rural population. The quantitative nature of the study using structured questions with “yes” or “no” answers also did not probe the reasons why patients hold a certain outlook about stroke. The duration of hypertension of the hypertensive patients was also not considered during the data collection process.

## Conclusions

Most hypertensive patients were aware of stroke, but many patients could not correctly identify the risk factors and warning signs of a stroke. Stroke prevention practices, while present, were also sub-optimal. A few misconceptions remain. The common symptoms identified for stroke were sudden onset of weakness/numbness of limbs and fainting. Poor recognition of other symptoms might contribute to low threat perception of stroke. Knowledge of a few risk factors are present, and notably, hypertension followed by diabetes mellitus. The factors contributing to choosing to take a person to the hospital (if they witness him/her with stroke symptoms) are modifiable. This emphasizes the need to organize awareness campaigns at the national level to increase knowledge regarding risk factors, which will benefit the community at large.

## Appendices

Questionnaire

Age:                                                                            Gender:  Male/Female

Religion: Muslim/Christian/Hindu                             Education: Primary/Secondary/ Higher

Income: Lower/Middle/Higher                                  Marital Status: Single/Married

**Table 6:** Questionnaire

Questions	Yes	No
Do you have any Family History of Stroke:		
Do you have any History of Stroke in Neighbour:		
Do you have any Personally know someone with stroke		
Have you ever read/heard about a disease called stroke?		
Is stroke a disease of the brain?		
Do you think homeopathic treatment is beneficial for stroke?		
Is stroke contagious?		
Is stroke a hereditary disease?		
Do you think stroke is caused by ancestor’s sin?		
Do you think stroke can be prevented?		
Is stroke an old person disease?		
Which of the following are warning signs of stroke:		
Sudden onset of dizziness		
Sudden onset of loss of vision		
Sudden onset of headache		
Sudden onset of memory loss		
Sudden onset of weakness/numbness of arm/leg		
Sudden onset of fainting		
Sudden onset of double vision		
Which of the following are risk factors of stroke?		
High blood pressure		
Smoking		
Diabetes		
High Cholesterol		
Obesity		
Do you:		
Exercise:		
Consume fruits and vegetables		
Avoid fatty foods		
Take medicines regularly		
Check your blood pressure regularly		
Smoke		
What will you do if you happen to witness a person with stroke?		
Take him/her to the hospital		
Sprinkle water on face		
Wait for spontaneous recovery		
